# Temporal Topic Modeling to Assess Associations between News Trends and Infectious Disease Outbreaks

**DOI:** 10.1038/srep40841

**Published:** 2017-01-19

**Authors:** Saurav Ghosh, Prithwish Chakraborty, Elaine O. Nsoesie, Emily Cohn, Sumiko R. Mekaru, John S. Brownstein, Naren Ramakrishnan

**Affiliations:** 1 Department of Computer Science, Virginia Tech, Arlington, Virginia, USA; 2 Children’s Hospital Informatics Program, Boston Children’s Hospital, Boston, Massachusetts, USA; 3 Department of Pediatrics, Harvard Medical School, Boston, Massachusetts, USA; 4 Institute for Health Metrics and Evaluation, University of Washington, Seattle, Washington, USA; 5 Epidemico, Inc., Boston, Massachsuetts, USA; 6 Department of Epidemiology, Biostatistics and Occupational Health, McGill University, Montreal, Canada

## Abstract

In retrospective assessments, internet news reports have been shown to capture early reports of *unknown* infectious disease transmission prior to official laboratory confirmation. In general, media interest and reporting peaks and wanes during the course of an outbreak. In this study, we quantify the extent to which media interest during infectious disease outbreaks is indicative of trends of reported incidence. We introduce an approach that uses supervised temporal topic models to transform large corpora of news articles into temporal topic trends. The key advantages of this approach include: applicability to a wide range of diseases and ability to capture disease dynamics, including seasonality, abrupt peaks and troughs. We evaluated the method using data from multiple infectious disease outbreaks reported in the United States of America (U.S.), China, and India. We demonstrate that temporal topic trends extracted from disease-related news reports successfully capture the dynamics of multiple outbreaks such as whooping cough in U.S. (2012), dengue outbreaks in India (2013) and China (2014). Our observations also suggest that, when news coverage is uniform, efficient modeling of temporal topic trends using time-series regression techniques can estimate disease case counts with increased precision before official reports by health organizations.

Infectious diseases are a threat to public health and economic stability of many countries. Open source indicators (e.g., news articles^1^,^2^, blogs^3^, search engine query volume^4^,^5^,^6^,^7^, social media chatter^8^,^9^,^10^,^11^ and other sources^12^) are an attractive option for monitoring infectious disease progression, primarily due to their sheer volume and capacity to capture early signals of disease outbreaks, and in some cases, trends in population health-seeking behavior. However, most prior work in digital surveillance using open source indicators has targeted specific diseases, such as influenza^12^,^13^ and hantavirus pulmonary syndrome (HPS)^14^. Therefore, there is a need to develop generic frameworks that are applicable to multiple infectious diseases.

Official surveillance reports released by health organizations (e.g., CDC, WHO, PAHO) are published with a considerable delay of weeks, months or even a year. Therefore, traditional surveillance systems are not always effective at real-time monitoring of emerging public health threats. Unlike traditional surveillance data, informal digital sources, such as news media, blogs, and micro-blogging sites (Twitter) are typically available in (near) real-time. Proper mining of signals from these digital sources can effectively help in minimizing the time lag between an outbreak start and formal recognition of an outbreak, allowing for an accelerated response to public health threats. The gains in supplementing traditional surveillance using digital sources have been discussed in Nsoesie *et al*.^15^, Salathé *et al*.^16^,^17^ and Hartley *et al*.^18^.

Our key contributions are as follows. (i) We introduce EpiNews, a generic temporal framework for analyzing disease-related news reports using a supervised topic model. The supervised topic model discovers multiple disease topics of interest and their associated temporal trends of prominence in news media. (ii) EpiNews captures trends in disease progression, such as periodicity, peaks and troughs via temporal trends of disease topics in news media. (iii) When news coverage is adequate, EpiNews also estimates disease incidence before official reports by health agencies using time-series regression models interposed over the temporal trends of disease topics.

We validated our method against disease case count reports, as available from public health agencies in U.S., China, and India. Disease-related news articles were provided by HealthMap^19^, an internationally recognized, global disease alert system capturing outbreak reports from over 200,000 electronic news sources. EpiNews was evaluated on multiple outbreaks in the recent past, such as whooping cough in U.S. (2012)^20^, periodic outbreaks of avian influenza A(H7N9)^21^,^22^ and hand, foot, and mouth disease (HFMD) in China (2013 and 2014), periodic outbreaks of acute diarrheal disease (ADD) in India (2013 and 2014), major dengue outbreaks in China (2014)^23^ and India (2013). Our experiments indicate that EpiNews was successfully able to capture the dynamics of the mentioned outbreaks and estimate the case counts in many of these instances before official reports were published. However, inconsistent news coverage was found to adversely affect the performance of our approach.

## Materials and Methods

### Data sources

In this section, we discuss the data sources used to analyze the infectious disease outbreaks. We first describe the case count reports collected from public health agencies and complete our discussion about the HealthMap data used in this study.

#### Disease case counts

For each country, we collected case count data corresponding to multiple diseases over a certain time period. In Table 1, we show the disease names (along with methods of transmission), health agencies from which case counts were collected, time period over which case counts were obtained and temporal granularity (daily, monthly, weekly or yearly) of the obtained case counts corresponding to each country.

#### HealthMap

Disease-related news articles were found to be indicative of infectious disease outbreaks^14^. We collected such articles related to the mentioned diseases in Table 1, for each country under consideration, from HealthMap. The HealthMap corpus is a publicly available database from which we collected the disease-related articles, reported during the time period of interest. Each article contains the reported date and the corresponding location information in the form of (lat, long) co-ordinate pairs. We converted the location co-ordinates to location names (country, state) via reverse geocoding. Reverse geocoding is defined as the process of finding a readable address or place name for a given (lat, long) pair. For example, (26.562851, −81.949532) was converted to (*United States, Florida*) after reverse geocoding. Each HealthMap article was passed through a series of preprocessing steps. For China, majority (87.94%) of the articles were published in either Traditional Chinese or Simplified Chinese. We translated the textual content of these articles to English for ease of analysis using Google translate (https://translate.google.com/). Because of the unavailability of ground truth for these articles, we couldn’t validate the performance of Google translate in this context. However, Google translate is one of the state-of-the-art commercial machine translations used today. Recent advances in deep learning and neural machine translation have made it a reliable tool for Chinese-to-English translation. For more details, see http://www.androidauthority.com/google-translate-machine-learning-chinese-718813/. Prior research^25^,^26^,^27^ on Chinese sentiment analysis has shown that using Google translate to translate Chinese reviews into English reviews improves the sentiment classification performance. In Pak *et al*.^26^, Google translate also yielded better results for the sentiment classification task in comparison to another commercial machine translation service named Yahoo Babelfish (http://babelfish.yahoo.com/).

### EpiNews

**Figure 1:**
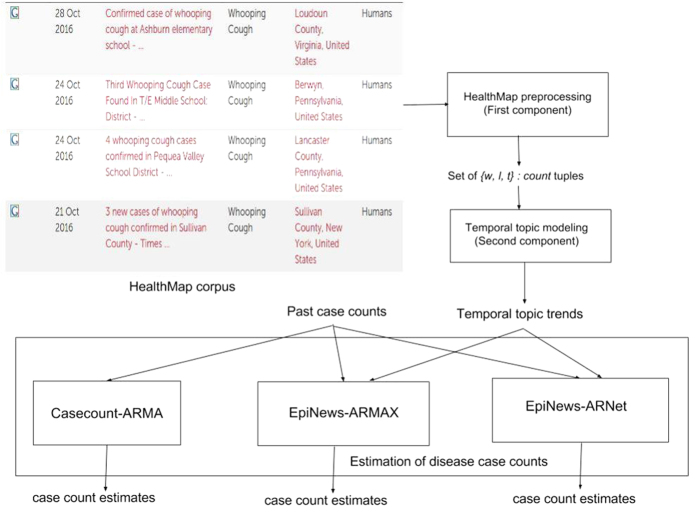
Flow chart depicting the sequential modeling process in EpiNews.

In this section, we describe in details the components of our proposed framework EpiNews. In Fig. 1, we show a flowchart depicting the sequential modeling process in EpiNews. The first component is the HealthMap preprocessing step which takes the HealthMap corpus as input and outputs the set 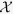 where each element represents a three-dimensional tuple of the form {*word(w*), *location(l*), *timepoint(t*)}:*count*. The second component, referred to as temporal topic modeling, is used to extract temporal topic trends from 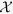. The final component, referred to as EpiNews-ARNet, is responsible for generating estimates of disease case counts using past available case counts and temporal topic trends extracted by the supervised topic model.

#### HealthMap preprocessing

The first component of EpiNews deals with the preprocessing of HealthMap articles through a series of preprocessing steps, such as removal of non-textual elements, tokenization^28^,^29^, lemmatization^30^ and removal of stop words via BASIS Technologies’ Rosette Language Processing (RLP) tools^31^,^32^. For more details on these steps, see Supplementary Section ‘HealthMap preprocessing’. The set of unique words in these processed articles were found to contain general- (e.g., *cold, contagious, nausea, blood, food-borne, waterborne, sanitation*) as well as specific- (e.g., *rabies, whooping, h7n9, dengue, salmonella, malaria*) disease related terms. In Table 2, we show country-wise distribution of the total number of HealthMap news articles along with unique words and location names extracted from all the corresponding articles.

Following Rekatsinas *et al*.^14^, the processed corpus for each country was transformed to a collection of tuples of the form {*w, l, t*}:*count*, where *count* is the number of news articles mentioning the word *w* associated with the location *l* and time point *t* in the tuple. For this transformation, we assumed that for each country, each processed article consists of words from a vocabulary *V*, corresponds to a discretized time window *t* ∈ {1, 2, …, *T*} and is geotagged with a location *l* from a set of locations *L* in the country. For China, disease case counts were available on a monthly granularity and as such each time point *t* represents a period of 1 month. However, for diseases in U.S. and India, case counts were obtained on a weekly basis and as such time point *t* represents a period of 1 week or more specifically, epidemiological week (hereafter referred to as epi week). For example, the tuple (*salmonella*, (*United States, Kansas*), 2013-10-06):9 denotes that the word *salmonella* was mentioned in 9 articles referring to the state of *Kansas* in U.S. over the epi week extending from 6^*th*^ October 2013 to 12^*th*^ October 2013. For each country, let *N*_*l*_ represent the collection of tuples for each location *l* ∈ *L* and 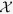 denote the set of all tuple collections *N*_*l*_ until time point *T*. This transformed set 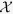 was analyzed to extract the temporal trends of disease topics as discussed in the following section. Both *N*_*l*_ and 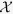 were updated for each country, as we proceed along the time window.

#### Temporal topic modeling

The second component of EpiNews deals with the topic and pattern discovery problem. The set 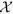 of all tuple collections *N*_*l*_ can be treated as a three-dimensional matrix of size *V* × *L* × *T* where the dimensions are represented by words (size *V*), locations (size *L*) and time points (size *T*). Each element *x*_*w*,*l*,*t*_ in 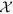 represents the total number of articles mentioning the word *w (w* ∈ *V*) referring to location *l (l* ∈ *L*) over the time point *t (t* ∈ 1, 2, 3, …, *T*). We assume that each entry in a non-zero element *x*_*w*,*l*,*t*_ of 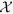 is associated with a latent disease topic and therefore, such hidden disease topics can be modeled in terms of three dimensions of 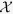. Our goal is to extract the hidden disease topics and their corresponding associations with each dimension of 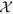. Following previous literature on topic models^14^,^33^,^34^,^35^, we implemented a supervised temporal topic model for this purpose. We supervise the discovery process of each disease topic by providing a set of prior words (also called seed words)^35^. These seed words are user-provided prior knowledge of each infectious disease and they encourage the topic model to find evidence of these disease topics in the HealthMap corpus. This supervised method helps in improving the discovery of word co-occurrences within each topic as the model tends to discover words that are related to the words in the seed set. Additionally, we model time and location jointly^14^ with the word co-occurrence patterns. This enables tracking of temporal and spatial patterns of these disease topics in the news. For more details on the supervised topic model, see Supplementary Section ‘Generative process of the supervised topic model’.

The supervised topic model takes 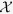 as input, discovers *K* disease topics and decomposes 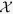 into four two-dimensional matrices as shown below. Each two-dimensional matrix represents the association between the discovered disease topics and the dimensions in 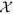.

- *ξ*: A *K* × *T* matrix where each row represents a discrete probability distribution over the time points (1, 2, 3, …, *T*) for a specific topic *z* ∈ 1, 2, 3, …, *K*. Each row of *ξ (ξ*_*z*_) represents the temporal topic trends or distribution for the disease topic *z* ∈ 1, 2, 3, …, *K*.
- *ϕ*^*s*^: A *K* × *S* matrix where each row represents a discrete probability distribution over the set *S* of seed words for a specific topic *z* ∈ 1, 2, 3, …, *K. ϕ*^*s*^ is hereafter referred to as the seed topic distribution.
- *ϕ*^*r*^: A *K* × *V* matrix where each row represents a discrete probability distribution over the set of regular words for a specific topic *z* ∈ 1, 2, 3, …, *K*. The set of regular words refers to all the words in vocabulary *V* including the seed words. *ϕ*^*r*^ is hereafter referred to as the regular topic distribution.
- *θ*: A *L* × *K* matrix where each row represents a discrete probability distribution over *K* topics for a specific location *l* ∈ *L*.

For more details on *ξ, ϕ*^*s*^, *ϕ*^*r*^ and *θ*, see Supplementary Section ‘Generative process of the supervised topic model’. Inference in this probabilistic model is conducted as follows. To compute the output parameters *θ, ϕ*^*r*^, *ϕ*^*s*^ and *ξ* in the supervised topic model given input observed data 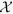, we need to solve an inference problem. In topic models, exact computation is intractable^33^ and thus we are interested in approximate inference of the model parameters. Since collapsed gibbs sampling^36^,^37^,^38^ is a straight-forward, easy to implement, and unbiased approach that converges rapidly to a known ground-truth, it is typically preferred over other possible approaches^33^,^39^ in large scale applications of topic models^14^,^37^,^40^. Thus we used collapsed gibbs sampling as the inference scheme for the supervised topic model. For more details on the inference process, see Supplementary Section ‘Inference via collapsed gibbs sampling’.

To apply the model in practice, seed words for each disease topic were extracted by examining the content of a subset of news articles mentioning the disease. Additionally, following similar techniques as in Chakraborty *et al*.^13^, we also examine a number of expert websites, such as CDC and WHO, to identify the most important keywords for a particular disease. Considering space limitations, seed words used in this study are shown in Supplementary Tables 1, 2 and 3 corresponding to diseases in U.S., China, and India respectively.

#### Estimation of disease case counts

The final component of EpiNews is concerned with estimation of disease case counts using relevant information such as past case counts and temporal topic trends (*ξ*). Let *D* be the disease of interest. Without loss of generality, let the *z*^*th*^ disease topic corresponds to *D*. Furthermore, let *S*_*D*,*T*_ denotes case counts of *D* and *ξ*_*z*,*T*_ denotes temporal trend value for *z*^*th*^ disease topic at a time point *T*. In general, reports of case counts published by health organizations are delayed (see Chakraborty *et al*.^13^, Wang *et al*.^41^) and hence, at time point *T* case counts are available only till *T′* < *T* with a delay *δ* = *T* *−* *T′*. However, temporal topic trend values 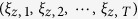 are available till T. Hence, we can formally define the case count estimation problem as estimating *S*_*D*,*T*_ using past case counts (*S*_*D*_) available till *T′* and temporal topic trends (*ξ*_*z*_) available till *T*. In general, disease case counts have a publication delay of 1 time point (*T′* = *T* − 1) and hence, estimating *S*_*D*,*T*_ at *T* is equivalent to 1-step ahead estimation.

#### EpiNews-ARNet

For 1-step ahead case count estimation, we used a regularized version of autoregressive model with external input variables (ARX) where external input variables are represented by the temporal topic trends (*ξ*_*z*_). We used Elastic Net^42^ as the regularization model in ARX. This estimating component of EpiNews is designated as EpiNews-ARNet and defined below in equation (1).


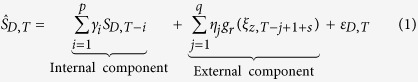


where, 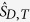 is the estimated case count for disease *D* at time point *T* and *γ*_*i*_, *η*_*j*_ are the regression coefficients fitted using Elastic Net constraints as given below in equation (2).


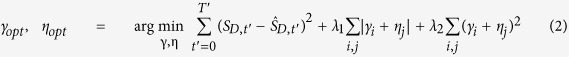


where, *λ*_1_ and *λ*_2_ are the regularization coefficients for the *L*1 and *L*2 components of Elastic Net, respectively. The Elastic Net combines the properties of Least Absolute Shrinkage and Selection Operator (LASSO)^43^,^44^ and Ridge regression^44^ models. This combination allows for learning a sparse model like LASSO, while still maintaining the regularization properties of Ridge. If *λ*_1_ equals to 0, equation (2) equates to a Ridge estimator. On the other hand, if *λ*_2_ equals to 0, equation (2) corresponds to a LASSO estimator.

There are broadly two components to equation (1) which captures different signals about the diseases as follows. (i) **Internal component (*****p***): This component is an autoregressive model that captures the signal embedded in past case counts and thus describes a delayed model. *p* indicates the order of autoregression. (ii) **External component (***q, r, s*): This component can also be thought of as an autoregressive component over the temporal topic trends (*ξ*_*z*_) where *q* is the number of time points to look back. The temporal topic trends are subjected to two additional transformations as follows. (a) **Shift indicator (*****s***): Often, the incidence of news reports is not concurrent with the incidence of diseases, as recorded in the case counts. EpiNews-ARNet incorporates this information by shifting the temporal topic trend value *ξ*_*z*,*T*_ by *s* steps. The shift can be positive (indicating a lagging trend), negative (indicating a leading trend) or zero (indicating a co-incident trend). (b) **Rolling transformation (***r*): Disease case counts (*S*_*D*_) do not follow a strictly linear relationship with temporal topic trends (*ξ*_*z*_). One of the simplest methods is to detrend the signals using difference of trend values instead of absolute values. However, our experiments showed that such transformations using a single time point often lead to unstable estimates. As such, we define a rolling transformation *g* over a window length *r* given below in equation (3).


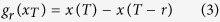


Essentially, such transformations aim to capture the changes in trend values over a period and were found to be more indicative than absolute values. We ran a cross-validation step to find the optimal (*p, q, r, s*) parameters.

#### Converting temporal topic trends to sampled case counts

We described EpiNews-ARNet using the temporal topic trends or distribution (*ξ*_*z*_) as the external input variables. It is to be noted that the disease case counts (*S*_*D*_) and the temporal topic distribution (*ξ*_*z*_) are typically at different numerical scales since values in a distribution range from 0 to 1. To improve numerical stability we converted the temporal topic distributions to estimated case counts using multinomial sampling^45^ over the time range. In multinomial sampling, samples are drawn from a multinomial distribution^45^. The case counts estimated via multinomial sampling from the temporal topic distributions are hereafter referred to as sampled case counts. To calculate the sampled case counts (Ξ_*D*_) for disease *D*, the corresponding temporal distribution *ξ*_*z*_ for *z*^*th*^ topic was used as the multinomial distribution and the total number of case counts available till *T′* < *T* at *T* (due to delay in reporting of case counts) was used as the number of samples to be drawn from the distribution. See Algorithm (1) for more details.


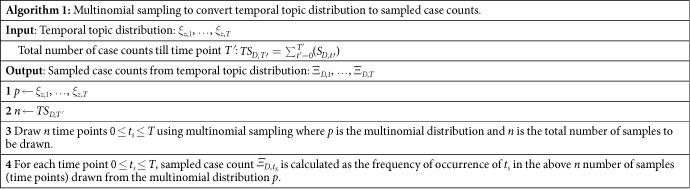


## Results

In this section, we present an empirical evaluation of our proposed framework EpiNews. We first evaluated the disease topics discovered by the supervised topic model. Next, we analyzed whether the temporal topic trends (*ξ*) extracted by the supervised topic model are able to capture disease dynamics - including seasonality, abrupt peaks and troughs. Finally, we evaluated the quality of case counts estimated by EpiNews-ARNet against the actual disease case counts.

### Disease topic discovery

To evaluate the discovered disease topics, we looked at the words having higher probabilities in the seed topic distributions (*ϕ*^*s*^) and regular topic distributions (*ϕ*^*r*^). Considering space limitations, we present the analysis of *ϕ*^*s*^ and *ϕ*^*r*^ in Supplementary Tables 1, 2 and 3 corresponding to disease topics in U.S., China and India respectively. For each country, both *ϕ*^*s*^ and *ϕ*^*r*^ were extracted from HealthMap data spanning over the entire time period shown in Table 1. For each disease topic (*z*), we show the seed words and their corresponding probabilities (sorted in descending order) in the seed topic distribution 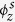. Seed words having higher probabilities in 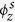 serve as informative prior words in the topic discovery process as they are mentioned frequently in news articles related to the *z*^*th*^ disease topic. For example, seed words such as *food, salmonella, product, fda, drug, contamination* serve as informative prior words for the discovery of salmonellosis topic in U.S. since they have higher probabilities in the seed topic distribution (see Supplementary Table 1). On the other hand, seed words such as *enteritidis, newport* provide less prior information due to their low probability values in the seed topic distribution. To understand how the supervised topic model discovers words from the HealthMap corpus related to these input seed words, we also show some of the regular words having higher probabilities in the regular topic distribution 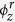. For a particular disease topic, these regular words with higher probabilities are mentioned frequently in news articles related to that disease and also capture different aspects (causes and clinical symptoms, methods of transmission, etc.) of the disease that the topic represents. For example, in Supplementary Table 1 we show these regular words (having higher probabilities in the regular topic distribution 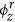) for the salmonellosis topic in U.S. Words such as *diarrhea*, nausea, *vomit* are related to clinical symptoms of salmonellosis. On the other hand, words such as *eat, contaminated, restaurant, meat, beef* are related to causes of salmonellosis.

### Detection of outbreak patterns

**Figure 2:**
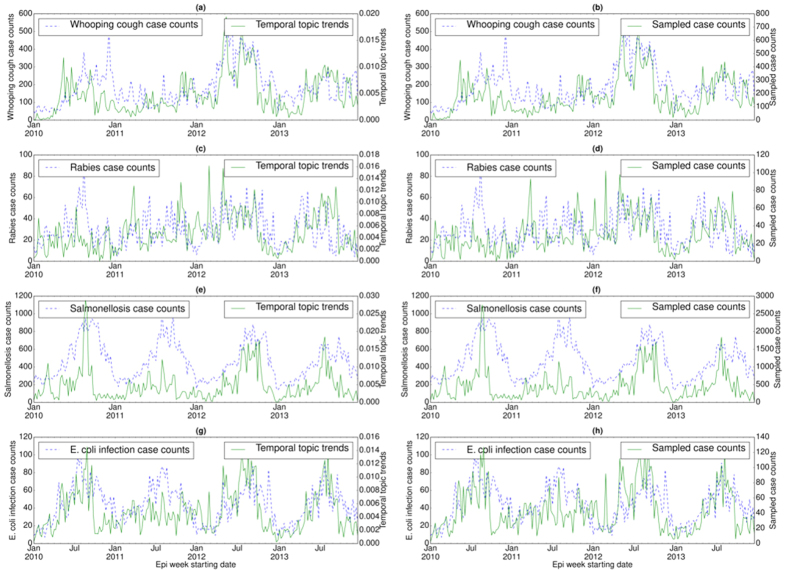
Correlation between disease case counts and temporal topic distributions or trends (*ξ*_*z*_) extracted by EpiNews for (**a**) whooping cough, (**c**) rabies, (**e**) salmonellosis, and (**g**) *E. coli* infection in U.S. Along with the temporal topic trends (*ξ*_*z*_), we also showed the correlation between disease case counts and sampled case counts (generated by multinomial sampling from temporal topic trends) for (**b**) whooping cough, (**d**) rabies, (**f**) salmonellosis, and (**h**) *E. coli* infection. Note, the sampled case counts and disease case counts share almost similar numerical range. However, the temporal topic trend values are at different numerical range (ranging from 0 to 1) with respect to the disease case counts.

**Figure 3:**
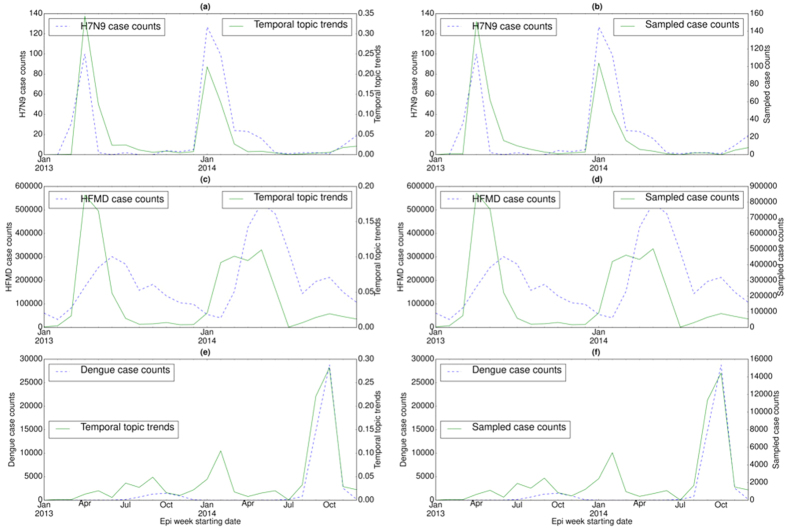
Correlation between disease case counts and temporal topic distributions or trends (*ξ*_*z*_) extracted by EpiNews for (**a**) H7N9, (**c**) HFMD, and (**e**) dengue in China. Along with the temporal topic trends (*ξ*_*z*_), we also showed the correlation between disease case counts and sampled case counts (generated by multinomial sampling from temporal topic trends) for (**b**) H7N9, (**d**) HFMD, and (**f**) dengue. Note, the sampled case counts and disease case counts share almost similar numerical range. However, the temporal topic trend values are at different numerical range (ranging from 0 to 1) with respect to the disease case counts.

**Figure 4:**
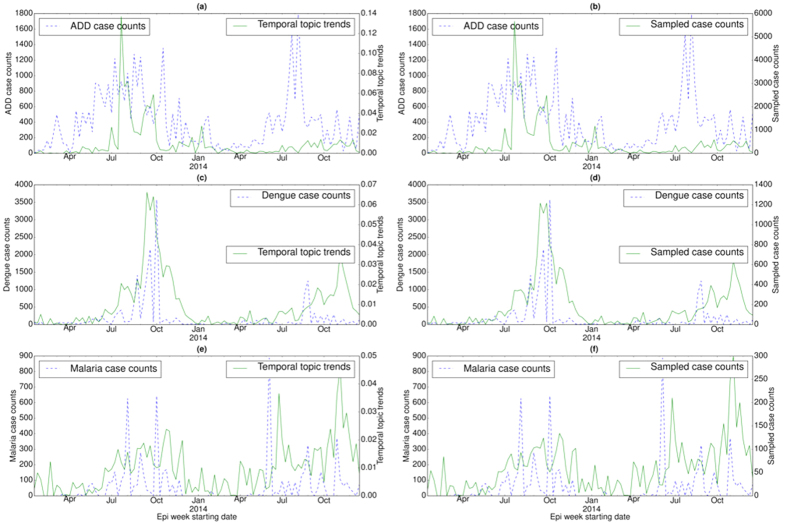
Correlation between disease case counts and temporal topic distributions or trends (*ξ*_*z*_) extracted by EpiNews for (**a**) ADD, (**c**) dengue, and (**e**) malaria in India. Along with the temporal topic trends (*ξ*_*z*_), we also showed the correlation between disease case counts and sampled case counts (generated by multinomial sampling from temporal topic trends) for (**b**) ADD, (**d**) dengue, and (**f**) malaria. Note, the sampled case counts and disease case counts share almost similar numerical range. However, the temporal topic trend values are at different numerical range (ranging from 0 to 1) with respect to the disease case counts.

We also examined the temporal distribution or trends (*ξ*_*z*_) for each disease topic (*z*) in a specific country (Figs 2, 3 and 4) and their correlations with the disease case counts. For each country, temporal topic trends (*ξ*_*z*_) were extracted from HealthMap data spanning over the entire time period shown in Table 1. We made several important observations as follows.

#### Disease seasonality

In U.S., case counts of salmonellosis and *E. coli* infection exhibit strong periodic outbreaks, both peaking during the summer (see Fig. 2(e) and (g)). Temporal topic trends extracted by EpiNews were able to capture the periodicity of these two diseases, particularly periodic outbreaks of salmonellosis and *E. coli* infection in 2010, 2012 and 2013. However, during 2011, temporal topic trends failed to monitor the peak season properly though they show a tendency to increase during summer. For salmonellosis in 2013, the temporal topic trends captured the major peak of the outbreak at the start of the season while failing to capture the seasonal activity towards the end. For rabies, although the topic trends captured the general characteristics it failed to detect some major outbreaks, such as the outbreak in the summer of 2010 (see Fig. 2(c)).

In China, H7N9 and HFMD case counts exhibit strong periodic outbreaks, with H7N9 peaking during the winter and HFMD peaking during the summer (see Fig. 3(a) and (c)). For H7N9, temporal topic trends extracted by EpiNews were able to detect the seasonal outbreaks during March–April 2013 and January–February 2014. However, for HFMD, peaks in temporal topic trends precede the peaks in case counts during the summer of 2013 and 2014 respectively. Therefore, temporal topic trends for HFMD exhibit a negative shift (leading indicator) with respect to the case counts.

In India, case counts of ADD exhibit periodic outbreaks, peaking during the summer of 2013 and 2014 (see Fig. 4(a)). Temporal topic trends detected the seasonal outbreak in the summer of 2013 but failed to capture the outbreak in the summer of 2014.

#### Sudden peaks/troughs

In U.S., whooping cough outbreaks do not exhibit yearly periodicity unlike salmonellosis and *E. coli* infection (see Fig. 2(a)). There was a major outbreak of whooping cough during the summer of 2012 and EpiNews detected this sudden increase (peak) in case counts by displaying higher topic trends during the entire period of the outbreak. EpiNews also did not detect outbreaks during periods (summer of 2011 and 2013) known to have low incidences (troughs) of whooping cough by displaying lower topic trends, suggesting low false alarm rate.

In China and India, dengue case counts exhibit seasonal outbreaks with peaks in case counts appearing during the months of September and October. However, China experienced a severe dengue outbreak in 2014^23^ in comparison to the outbreak in 2013 with the peak value of case counts exceeding 25,000 in the month of October (see Fig. 3(e)). Temporal topic trends detected this sudden massive increase in case counts by displaying a sharp spike during the outbreak period. India also experienced a large dengue outbreak in 2013 with the peak value of case counts exceeding 3,000 during a particular epi week in October (see Fig. 4(c)). EpiNews was able to detect this outbreak by displaying higher topic trends during the peak period. Malaria case counts in India exhibit irregular outbreaks or peaks (see Fig. 4(e)). EpiNews was successful in capturing majority of these outbreaks though it failed to detect some major peaks, such as the peak during the month of June 2014.

#### Sampled case counts

Along with the temporal topic trends (*ξ*_*z*_), we also showed the corresponding sampled case counts (Ξ_*D*_) generated via multinomial sampling (see Algorithm (1)) from *ξ*_*z*_ for a disease *D* in Figs 2(b,d,f and h), 3(b,d and f) and 4(b,d and f). The figures show that the sampled case count values share similar numerical range as the disease case counts while maintaining shapes of the temporal topic trends. On the other hand, the temporal topic trend values are at different numerical range (ranging from 0 to 1) with respect to the case counts.

### Estimating case counts

As official reports of case counts by health agencies are usually lagged by a single time point (week or month), reliable early estimates of disease incidence can facilitate the allocation of public health resources to enable effective control measures. Therefore, we aim to perform 1-step ahead estimation of disease case counts starting from a particular time point. For the purpose of experimental validation, we used historical HealthMap data over a certain time period as the static training set in a specific country (referred to as the static training period) and progressively utilized the remaining time points as the evaluation period over which we evaluated the case count estimates of EpiNews-ARNet. To estimate case counts at a particular time point *T* within the evaluation period, we utilized HealthMap data from *t* = 0 up to *t* = *T* and extracted disease topics using the supervised topic model. The disease case counts at *T* were next estimated using past case counts available up to *t* = *T′ (T′* = *T* − 1) and temporal topic trends (or, sampled case counts) available up to *t* = *T*. In Table 3, we show the total time period of study, static training period and the evaluation period for each country.

#### Baselines

For the task of 1-step ahead estimation, we compared the performance of EpiNews-ARNet against 2 baseline methods, namely EpiNews-ARMAX and Casecount-ARMA. In Casecount-ARMA, we fitted an autoregressive-moving-average model (ARMA^46^) over past disease case counts to generate case count estimates. Casecount-ARMA doesn’t use any information related to temporal topic trends (*ξ*_*z*_). However, in case of EpiNews-ARMAX, we used an autoregressive—moving-average model with external input variables (ARMAX^46^) where external input variables incorporate the information embedded in temporal topic trends. For more details on the baseline methods, see Supplementary Section ‘Baseline methods for case count estimation’. We also compared temporal topic trends against sampled case counts (generated by multinomial sampling from the temporal topic trends) as the external input variables, for the applicable methods EpiNews-ARNet and EpiNews-ARMAX.

#### Evaluation

We evaluated the case count estimates of each method over the evaluation period by comparing them against the actual case counts using normalized root-mean-square error (NRMSE). In Table 4, we present a comparative performance evaluation of the methods for 1-step ahead estimation in terms of NRMSE values corresponding to diseases in U.S., China and India respectively. Table 4 provides multiple insights as follows. (i) EpiNews-ARNet with sampled case counts as external variables is the best performing method achieving lowest NRMSE values for majority (8 out of 10) of the {country, disease} combinations. (ii) Two exceptions are {China, HFMD} and {U.S., *E. coli* infection} where EpiNews-ARNet and EpiNews-ARMAX with temporal topic trends as external variables achieve lowest NRMSE values respectively. (iii) Both EpiNews-ARNet and EpiNews-ARMAX perform better overall with sampled case counts as external variables than temporal topic trends. (iv) For none of the {country, disease} combinations, Casecount-ARMA is able to achieve lowest NRMSE values indicating the significance of incorporating temporal topic trends or sampled case counts as external variables for estimating case counts.

## Discussion

In this paper, we studied the problem of monitoring and estimating outbreaks of multiple infectious diseases using disease-related online news reports obtained from HealthMap. We introduced EpiNews, a novel and generic temporal framework that combines supervised temporal topic models with time-series regression techniques to monitor and estimate disease incidence. Experimental results demonstrate that EpiNews is able to capture the time varying incidence of multiple diseases via temporal topic trends. Our experiments also illustrate that EpiNews can estimate disease incidence 1-step ahead with increased accuracy using information from temporal topic trends.

EpiNews uses online news reports as the sole data source to capture disease dynamics during outbreaks. Therefore, it is generic in the sense that it is not tailored to a particular disease or class of diseases. Moreover, the set of diseases selected for each country represent a diversity of transmission pathways as shown in Table 1. Hence, the applicability of EpiNews to these diverse sets of diseases as demonstrated in this study showcases the potential generalizability of our approach to different class of diseases.

Temporal topic trends extracted by EpiNews from HealthMap news reports successfully captured dynamics of multiple outbreaks, such as whooping cough in U.S. during summer of 2012, periodic outbreaks of salmonellosis and *E. coli* infection in U.S., periodic outbreaks of H7N9 and HFMD in China, dengue outbreaks in India (2013) and China (2014). However, there are certain deviations where temporal topic trends could not monitor the trends in disease outbreaks properly. We posit that such deviations are a factor of multiple effects as follows. (i) Firstly, news media coverage during disease outbreaks is driven by interest. News coverage for certain diseases can be inconsistent over time. For salmonellosis and *E. coli* infection outbreaks in 2010, 2013 and 2014 (see Fig. 2(e) and (g)), the temporal topic trends capture the outbreak at the start of the season. However, as the outbreak season progresses, the temporal topic trends are unable to capture the outbreak dynamics accurately. This indicates that news media coverage is generally high during the start of a disease outbreak. However, we observe a decline in news media interest as the outbreak season progresses. (ii) Secondly, for diseases with low public interest, the coverage can be low even there is an ongoing disease outbreak. E.g., in case of the ADD outbreak in 2014 (see Fig. 4(a)), we observe no coverage in news media (lack of activity in temporal topic trends) even though the outbreak occurred on a massive scale. (iii) Finally, our framework is heavily reliant on news corpora and does not account for possible reporting errors. As such, articles with missing or incomplete textual content can affect the performance of our framework. E.g., in case of salmonellosis and *E. coli* infection outbreaks in 2011, the rise in temporal topic trends is comparatively lower during the outbreak period (see Fig. 2(e) and (g)) in comparison to the outbreaks in 2010, 2012, and 2014.

EpiNews supports monitoring and also 1-step ahead estimation of disease case counts with increased precision. Table 4 shows that EpiNews-ARNet yields lowest NRMSE values for all the diseases when compared to the baseline method Casecount-ARMA. This implies that incorporating information from temporal topic trends via EpiNews-ARNet results in improved estimation of case counts. It is also to be noted that EpiNews-ARNet with sampled case counts as external variables achieves lower NRMSE for most of the diseases than the variant using temporal topic trends. This validates our claim that using sampled case counts instead of actual topic trends as the external variables adds numerical stability to EpiNews-ARNet.

The performance of EpiNews-ARMAX is comparable to EpiNews-ARNet for diseases in U.S. However, for diseases in China and India, EpiNews-ARNet significantly outperforms EpiNews-ARMAX. In China and India, both disease case counts and temporal topic trends (or, sampled case counts) are characterized by sharp peaks during the outbreak period (see Figs 3 and 4). EpiNews-ARMAX performs poorly in such scenarios (see Table 4) in comparison to EpiNews-ARNet, mainly due to the unstable behavior of the ARMAX model when it comes to handling sharp gradients in input case counts or temporal topic trends. However, outbreak periods for diseases in U.S. are characterized by flat peaks with slow rise and fall (see Fig. 2). Therefore, EpiNews-ARMAX achieves comparable performance to EpiNews-ARNet, even performing better for *E. coli* infection. Therefore, we conclude that both EpiNews-ARMAX and EpiNews-ARNet are preferred approaches for estimating case counts of diseases characterized by flat outbreak peaks with slow rise and fall. However, when disease outbreaks exhibit sharp peaks, we recommend selecting EpiNews-ARNet for reliable estimation of case counts.

**Figure 5:**
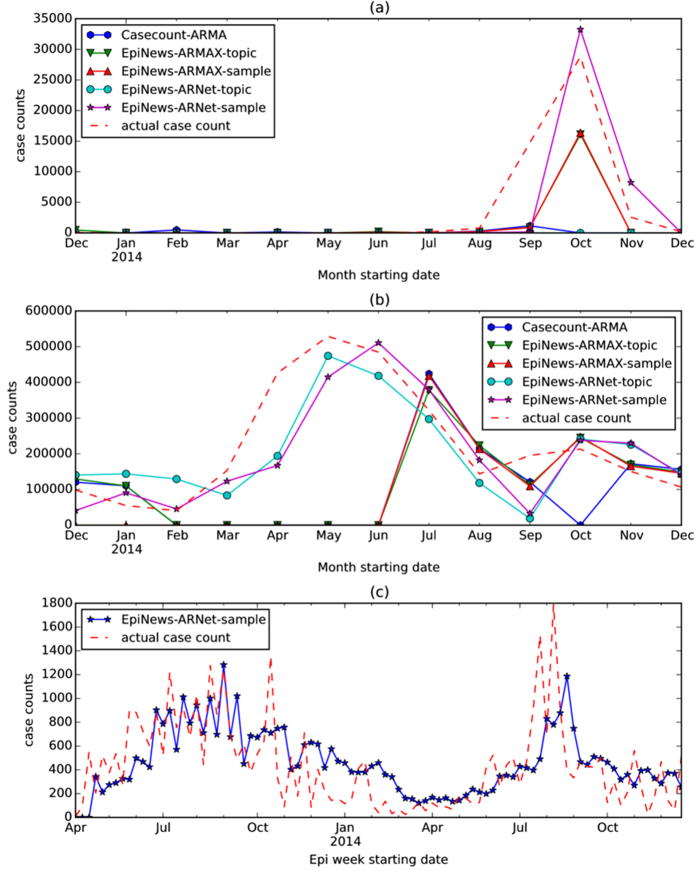
Temporal correlation between actual case counts and case counts estimated by the methods Casecount-ARMA, EpiNews-ARMAX and EpiNews-ARNet corresponding to (**a**) dengue and (**b**) HFMD in China. In (**a**) and (**b**), EpiNews-ARMAX -topic and EpiNews-ARNet -topic use temporal topic trends as external variables. On the other hand, EpiNews-ARMAX -sample and EpiNews-ARNet -sample use sampled case counts as external variables. In (**c**), we showed the temporal correlation between actual case counts and case counts estimated by EpiNews-ARNet -sample corresponding to ADD in India.

For dengue and HFMD in China, EpiNews-ARNet shows considerable improvement on 1-step ahead estimation of disease incidence when compared to the baselines, specifically Casecount-ARMA (see Table 4). In order to have a clearer understanding of the improved performance of EpiNews-ARNet with respect to the baselines, we plotted the temporal correlation between actual case counts and case counts estimated by the methods in Fig. 5 corresponding to dengue and HFMD in China. It can be observed that EpiNews-ARNet with sampled case counts as external variables is able to estimate the peak in dengue case counts more accurately in comparison to the baselines (see Fig. 5(a)). For HFMD, EpiNews-ARNet with both topic trends and sampled case counts as external variables are able to estimate the peak in case counts, while the baselines fail to do so (see Fig. 5(b)). Casecount-ARMA’s inability to estimate the peaks in case counts for both dengue and HFMD implies that past case counts are not reliable indicators for estimating sudden increases or peaks in disease incidence and therefore, need to be augmented with disease signals from online news media for accurate estimation of outbreaks. However, inconsistent news coverage can adversely affect the timely estimation of outbreaks by EpiNews-ARNet as shown in Fig. 5(c). India experienced periodic outbreaks of ADD with peaks in case counts during the summer of 2013 and 2014. However, we observe a lack of news coverage (no peak in temporal topic trends) during the peak in 2014 compared to the peak in 2013 (see Fig. 4(a) and (b)). Therefore, the case count estimates generated by EpiNews-ARNet have a delayed peak with respect to the actual peak in case counts during the outbreak in 2014 (see Fig. 5(c)). This delayed peak is due to the internal component (*p*) in equation (1) which extracts information from past case counts. In overall, our results over a range of diseases and world regions suggest that monitoring progression of infectious diseases is feasible and disease incidence can be estimated with increased precision via efficient capturing of signals from online news media.

The effectiveness of online sources (news, tweets, search queries) to monitor and forecast the emergence and/or spread of diseases is an ongoing topic of debate, as evidenced by the community response to the study of Lazer *et al*.^47^. They demonstrated that Google Flu Trends (GFT) was overestimating influenza-like illness (ILI) case counts in CDC reports. However, when GFT was combined with lagged CDC data, the authors observed a substantial improvement in estimating the CDC counts. In EpiNews based models (EpiNews-ARNet and EpiNews-ARMAX), unlike GFT, we have combined the lagged (past) disease case counts with the temporal topic trends extracted from the HealthMap news corpus in order to generate reliable case count estimates. However, during outbreak periods, inconsistent news media coverage and possible reporting errors can hamper forecasting performance as lagged case counts are not helpful in such scenarios and we must rely on external news trends for forecasting. Therefore, given consistent media coverage, EpiNews based models have the capability to generate reliable case count estimates (see Fig. 5(a) and (b)). However, in scenarios where news media depict a lack of (or inconsistent) coverage (Fig. 5(c)), we can supplement the model by leveraging information from physical data sources, such as climatic attributes (temperature^48^, precipitation^49^, and humidity^50^). The main take-away conclusion from Lazer *et al*.^47^, applicable to our work as well, is that models based on machine learning, such as developed here, need to be constantly tuned/retrained to ensure that model drift can be detected and corrected.

## Additional Information

**How to cite this article:** Ghosh, S. *et al*. Temporal Topic Modeling to Assess Associations between News Trends and Infectious Disease Outbreaks. *Sci. Rep.* **7**, 40841; doi: 10.1038/srep40841 (2017).

**Publisher's note:** Springer Nature remains neutral with regard to jurisdictional claims in published maps and institutional affiliations.

## Supplementary Material

Supplementary Information

## Figures and Tables

**Table 1:** Disease names (along with routes of transmission), health agencies from which case counts were collected, time period over which case counts were obtained and temporal granularity (daily, monthly, weekly or yearly) of the obtained case counts corresponding to each country.

Country	Disease names (Methods of transmission)	Health agencies	Time period	Temporal granularity
U.S.	Whooping cough (airborne, direct contact)Rabies (zoonotic) Salmonellosis (food-borne)*E. coli* infection (waterborne, food-borne)	Project Tycho^24^ (https://www.tycho.pitt.edu/)	January 2010–December 2013	Weekly
China	H7N9 (zoonotic) HFMD (direct contact, airborne) Dengue (vector-borne)	National Health and Family PlanningCommission (http://en.nhfpc.gov.cn/)	January 2013–December 2014	Monthly
India	ADD (food-borne) Dengue (vector-borne)Malaria (vector-borne)	Integrated Disease SurveillanceProgramme (http://www.idsp.nic.in/)	January 2013–December 2014	Weekly

H7N9 stands for avian influenza A, ADD stands for acute diarrheal disease and HFMD stands for hand, foot, and mouth disease.

**Table 2:** Country-wise distribution of the total number of HealthMap news articles along with unique words and location names extracted from all the corresponding articles.

Country	Total number of HealthMap news articles	Total number of unique words	Total number of unique location names or (country, state) pairs
China	11,209	21,879	30
India	1,204	17,160	30
U.S.	9,872	59,687	51

**Table 3:** Total time period of study, static training period and the evaluation period for estimating disease case counts in each country.

Country	Total time period of study	Static training period	Evaluation period
U.S.	January 2010–December 2013	January 2010–December 2011	January 2012–December 2013
China	January 2013–December 2014	January 2013–March 2013	April 2013–December 2014
India	January 2013–December 2014	January 2013–November 2013	December 2013–December 2014

**Table 4:** Comparing the performance of EpiNews-ARNet against the baseline methods EpiNews-ARMAX and Casecount-ARMA for 1-step ahead estimation of disease case counts.

Country	Disease	Casecount-ARMA	EpiNews-ARMAX		EpiNews-ARNet	
			with temporal topic trends	with sampled case counts	with temporal topic trends	with sampled case counts
U.S.	*Whooping* cough	0.584	0.577	0.582	0.583	**0.558**
	*Rabies*	0.875	0.888	0.886	0.877	**0.865**
	*Salmonellosis*	0.445	0.978	0.450	0.441	**0.430**
	*E. coli infection*	0.685	**0.657**	0.663	0.686	0.671
China	*H7N9*	1.096	0.850	0.888	1.027	**0.712**
	*HFMD*	1.574	1.524	1.538	**0.622**	0.626
	*Dengue*	1.076	0.639	0.634	1.094	**0.549**
India	*ADD*	1.226	1.285	1.119	0.844	**0.833**
	*Dengue*	0.966	1.086	1.021	1.073	**0.878**
	*Malaria*	1.060	1.062	1.047	1.016	**0.963**

Metric used for comparing the case counts estimated by the methods against the actual case counts is the normalized root-mean-square error (NRMSE).
