# Supplementary material for: Temporal Topic Modeling to Assess Associations between News Trends and Infectious Disease Outbreaks
Source: Sci Rep. 2017 Jan 19;7:40841. doi: 10.1038/srep40841 (PMC5244405; doi:10.1038/srep40841)
Supplement: Supplementary Information [file srep40841-s1.pdf]

Temporal Topic Modeling to Assess Associations between News Trends and Infectious Disease Outbreaks - Supplementary Information

Saurav Ghosh[1,*], Prithwish Chakraborty[1], Elaine O. Nsoesie[2, 3, 4], Emily Cohn[2], Sumiko R. Mekaru[3, 6], John S. Brownstein[2, 3, 5, 6], and Naren Ramakrishnan[1]

[1]Department of Computer Science, Virginia Tech, Arlington, Virginia, USA,
[2]Children's Hospital Informatics Program, Boston Children's Hospital, Boston, Massachusetts, USA,
[3]Department of Pediatrics, Harvard Medical School, Boston, Massachusetts, USA,
[4]Institute for Health Metrics and Evaluation, University of Washington, Seattle, Washington, USA,
[5]Department of Epidemiology, Biostatistics and Occupational Health, McGill University, Montreal, Canada,
[6]Epidemico, Inc., Boston, Massachsuetts, USA.
[*]sauravcsvt@vt.edu

ABSTRACT

Supplementary information for the manuscript entitled 'Temporal Topic Modeling to Assess Associations between News Trends and Infectious Disease Outbreaks'.

HealthMap preprocessing

Each HealthMap article was preprocessed using the following techniques.

1. **Extracting main textual content.** In the first step, we extracted the main textual content of each article using Dragnet[1] and Goose (`https://github.com/grangier/python-goose`), ignoring the non-textual elements such as images within the article.

2. **Tokenization and lemmatization.** Tokenization[2,3] is the process of segmenting a textual content into words, phrases, symbols or other meaningful elements commonly referred to as tokens. Lemmatization[4] is performed after tokenization and can be defined as the normalization process in which various inflected forms of a word are converted to the same underlying lemma so that they can be analyzed as a single term. For example, terms such as *travel*, *traveled*, *travels*, *TRAVEL*, *traveling*, *Travelling*, *travelling*, *travelled*, *Travel*, *Traveling* were converted to the same underlying lemma *travel*. In the second step, both tokenization and lemmatization were performed on the extracted textual content using BASIS Technologies' Rosette Language Processing (RLP) tools[5,6] to generate a set of unique words or phrases corresponding to each article.

3. **Uppercase to lowercase.** In the third step, we converted the uppercase letters in each extracted word to lowercase letters. For example, both the terms *Salmonella* and *salmonella* convey the same meaning, so they were converted to a single term *salmonella*.

4. **Removal of stop words.** In the final step, we removed all the stop words such as *in*, *by*, *of*, *at*, *all*, etc. from the set of unique words or phrases extracted from each article.

Validation of preprocessing steps. The preprocessing steps outlined above are traditional NLP preprocessing techniques used to extract a vocabulary of words from a corpus of articles. The tools used (Dragnet, Goose, Rosette) have been used in numerous text analytics research studies before and several existing studies have conducted validation experiments along the lines suggested by the reviewer. For instance, detailed discussions about the accuracy of Dragnet and Goose (used in the first step 'Extracting main textual content') on a test corpus (`https://github.com/seomoz/dragnet_data`) can be found in `https://moz.com/devblog/benchmarking-python-content-extraction-algorithms-dragnet-readability`
.The conclusion from these studies (and others) is that Dragnet performs better than Goose in terms of recall (example result: Dragnet : Goose = 87.4 % : 74.7 %) and speed (Dragnet : Goose = 70 Docs/sec : 11 Docs/sec), while Goose performs better

than Dragnet in terms of precision (Goose : Dragnet = 86.1 % : 84.6 %). In our study, as discussed earlier, we used both Dragnet and Goose to realize a combination of both precision and recall objectives.

The second step ‘Tokenization and lemmatization’ is executed using BASIS Technologies’ Rosette Language Processing (RLP) tools. For tokenization (separating textual content into its atomic elements, i.e. words), RLP tools provide support for 40 languages and use advanced statistical modeling to identify and separate each word. For more details, see https://www.rosette.com/function/tokenization/. Similarly, in case of lemmatization (finding the dictionary form of a word), RLP tools look at vocabulary, context and advanced morphological analysis to find the common dictionary form of a word resulting in more recall and better precision. For more details, see https://www.rosette.com/function/morphological-analysis/. RLP tools have been used in prior research[7,8] for studies on HealthMap data and therefore, can be considered as the state-of-the-art in preprocessing of text articles for probabilistic modeling as done in this manuscript.

Removing stop words do not cause any disease-related information loss (stop words refer to the most common words in English language, such as ‘the’, ‘and’, ‘or’, etc.). Uppercase to lowercase conversion does not cause any loss of information for modeling purposes.

Generative process of the supervised topic model

In this section, we will discuss in details the generative process of the supervised topic model. Before going into the details of the generative process, we will first define the notion of a topic in the supervised topic model. In unsupervised topic models[8–11] , each topic k is defined as a discrete probability distribution over all the words in the vocabulary V. In the supervised topic model, the notion of a topic is extended and defined as the convex combination of two discrete probability distributions: seed topic distribution and regular topic distribution[12] . The seed topic distribution can only generate words from the seed set S, and thus it is defined as a discrete probability distribution over only the words in the seed set S. On the other hand, the regular topic distribution has the freedom to generate any word including the seed words. So a regular topic is defined as a discrete probability distribution over all the words in the vocabulary V. Here we assume that each regular topic is associated with only one seed topic, i.e., there is a one-to-one correspondence between seed and regular topics.

Algorithm 1: Generative process of the supervised topic model

for *each topic* $k \leftarrow \{1,2,\ldots,K\}$ **do**
  Draw $\phi_k^r \sim \text{Dirichlet}(\beta^{(k)})$
  Draw $\phi_k^s \sim \text{Dirichlet}(\mu^{(k)})$
  Draw $\xi_k \sim \text{Dirichlet}(\gamma^{(k)})$
  Draw $\pi_k \sim \text{Beta}(1,1)$
for *each location* $l \in L$ **do**
  Draw $\theta_l \sim \text{Dirichlet}(\alpha^{(l)})$
  for *each entry* $i \in N_l$ **do**
    Draw topic $z_i \sim \text{Discrete}(\theta_l)$
    Draw indicator variable $x_i \sim \text{Bernoulli}(\pi_{z_i})$
    Draw $w_i \sim \begin{cases} \text{Discrete}(\phi_{z_i}^r) & \text{when } x_i = 0 \quad \text{// regular topic} \\ \text{Discrete}(\phi_{z_i}^s) & \text{when } x_i = 1 \quad \text{// seed topic} \end{cases}$
    Draw timestamp $t_i \sim \text{Discrete}(\xi_{z_i})$

The generative process of the supervised topic model is described in Algorithm (1). Given K disease topics, L locations and N_l for each $l \in L$, the supervised topic model uses location and topic specific discrete probability distributions to model the generation of word and time point in each entry of N_l. To generate each entry $i \in N_l$ for a location $l \in L$, we first sample a topic z_i ($z_i \in \{1,2,\cdots,K\}$) from the location-specific discrete probability distribution θ_l over K disease topics. To generate a word w_i, we choose either the seed topic distribution (ϕ^s) or the regular topic distribution(ϕ^r) corresponding to the sampled topic z_i. The indicator variable x_i sampled from Bernoulli (π_{z_i}) decides whether the word should be drawn from the seed topic distribution or the regular topic distribution. π_{z_i} is called the sampling probability for topic z_i. Once the distribution is chosen, the word w_i is generated from it. Finally, the time point t_i is drawn from the topic-specific discrete probability distribution ξ_{z_i} over the time points $\{1,2,\cdots,T\}$.

Choice of priors. ξ_k ($k \in \{1,2,....,K\}$) is drawn from an asymmetric Dirichlet prior[13,14] parameterized by a T-dimensional vector $\gamma^{(k)}$ as defined below in equation (1).

$$\gamma^{(k)} = [N_{k,1} + \gamma^{'}, N_{k,2} + \gamma^{'}, \cdots, N_{k,t} + \gamma^{'}, \cdots, N_{k,T} + \gamma^{'}] \tag{1}$$

where, $N_{k,t}$ is the sum of the *count* variable across those tuples ($\{w, l, t^{'}\}$: *count*) of $\mathscr{X}$ where the word w in the tuple is a seed word related to disease topic k, $t^{'}$ is equal to the time point t in equation (1) and l refers to any location in the set L. In other words, $N_{k,t}$ accounts for the occurrence of seed words related to topic k in $\mathscr{X}$ at time point t. Higher occurrence of seed words indicates higher prominence of topic k at time point t and vice versa. Therefore, asymmetric prior $\gamma^{(k)}$ is used to incorporate prior information into the supervised topic model regarding prominence of disease topic k at different time points. The hyperparameter $\gamma^{'}$ in equation (1) is an additional smoothing parameter that contributes a flat pseudocount to each component of $\gamma^{(k)}$. Additive smoothing is done to assign non-zero probabilities to those time points for which we have no prior information (zero occurrence of seed words) related to topic k.

θ_l is also associated with an asymmetric Dirichlet prior parameterized by a K-dimensional vector $\alpha^{(l)}$ as defined below in equation (2).

$$\alpha^{(l)} = [N_{l,1} + \alpha^{'}, N_{l,2} + \alpha^{'}, \cdots, N_{l,k} + \alpha^{'}, \cdots, N_{l,K} + \alpha^{'}] \tag{2}$$

where, $N_{l,k}$ is the sum of the *count* variable across those tuples ($\{w, l^{'}, t\}$: *count*) of $\mathscr{X}$ where the word w in the tuple is a seed word related to disease topic k, $l^{'}$ is equal to the location l in equation (2) and t can be any time point in the range $\{1, 2, \cdots, T\}$. In other words, $N_{l,k}$ accounts for the occurrence of seed words related to topic k in N_l. The hyperparameter $\alpha^{'}$ is the additional smoothing parameter that contributes a non-zero pseudocount to each component of $\alpha^{(l)}$. Additive smoothing is done to assign non-zero probabilities to those locations for which we have no prior information (zero occurrence of seed words) related to topic k.

Finally, seed topic distribution (ϕ^s) and regular topic distribution (ϕ^r) are drawn from symmetric Dirichlet priors[14] where each component of the parameter vectors $\mu^{(k)}$ (S-dimensional) and $\beta^{(k)}$ (V-dimensional) assumes the values of the hyperparameters $\mu^{'}$ and $\beta^{'}$ respectively, i.e., $\mu^{(k)} = [\mu^{'}, \mu^{'}, \cdots, \mu^{'}]$ and $\beta^{(k)} = [\beta^{'}, \beta^{'}, \cdots, \beta^{'}]$.

Choice of hyperparameters. A hyperparameter is defined as the parameter of a prior distribution. The hyperparameters $\alpha^{'}$, $\gamma^{'}$, $\beta^{'}$ and $\mu^{'}$ are set to $2/K$, 0.01, 0.01 and $1e-07$ respectively. These values are chosen heuristically, and an improved performance of the supervised topic model could be achieved via efficient hyperparameter optimization[14] . As suggested in Jagarlamudi et al.[12] , we set the sampling probability π_k to a constant value of 0.7 for each topic $k \in \{1, 2, \cdots, K\}$.

Inference via collapsed gibbs sampling

The key problem in the supervised topic model is posterior inference. This amounts to reversing the defined generative process and inferring the output (latent) parameters θ, ϕ^r , ϕ^s and ξ given the observed tuples in N_l. A standard approach of posterior inference in topic models is collapsed gibbs sampling[15] , a Markov Chain Monte Carlo (MCMC) method.

To estimate the model parameters θ, ϕ^r , ϕ^s and ξ via collapsed gibbs sampling, we need to compute the conditional probability distribution $\Pr(z_i = k|\mathbf{w}, \mathbf{t}, \mathbf{l}, \mathbf{z}_{-i}, \alpha^{(l)}, \beta^{(k)}, \mu^{(k)}, \gamma^{(k)})$ where z_i represents the topic assignment for the ith tuple or entry in N_l. $\mathbf{z}_{-i}$ represents the topic assignments for all entries in N_l except the ith entry. We have three scenarios as shown below.

- If word w_i in the ith entry of N_l is a regular word and k is a regular topic, then the conditional probability distribution is defined below in equation (3).

$$\begin{aligned}\Pr(z_i = k|\mathbf{w}, \mathbf{t}, \mathbf{l}, \mathbf{z}_{-i}, \alpha^{(l)}, \beta^{(k)}, \mu^{(k)}, \gamma^{(k)}) \propto & \frac{n_{w_i}^{k,-i} + \beta^{'}}{\sum_{v=1}^{V}(n_v^{k,-i} + \beta^{'})} \cdot \frac{m_{t_i}^{k,-i} + \gamma_{t_i}^{(k)}}{\sum_{t=1}^{T}(m_t^{k,-i} + \gamma_t^{(k)})} \cdot \frac{o_k^{l,-i} + \alpha_k^{(l)}}{\sum_{k^{'}=1}^{K}(o_{k^{'}}^{l,-i} + \alpha_{k^{'}}^{(l)})} \\ & \cdot \frac{(\sum_{v=1}^{V} n_v^{k,-i} + \beta^{'}) + \pi_k}{(\sum_{v=1}^{V} n_v^{k,-i} + \beta^{'}) + (\sum_{v=1}^{S} s_v^{k,-i} + \mu^{'}) + 2 \cdot \pi_k}\end{aligned} \tag{3}$$

- If word w_i in the ith entry of N_l is a regular word and k is a seed topic, then the conditional probability distribution $\Pr(z_i = k|\mathbf{w}, \mathbf{t}, \mathbf{l}, \mathbf{z}_{-i}, \alpha^{(l)}, \beta^{(k)}, \mu^{(k)}, \gamma^{(k)}) = 0$ since a regular word cannot be generated from any of the seed topic distributions.

- If word w_i in the ith entry of N_l is a seed word, then word w_i can be generated from either the seed topic k or the regular topic k. If word w_i is generated from a seed topic k, then the conditional probability distribution is defined below in equation (4). On the other hand, if word w_i is generated from a regular topic k, then the conditional probability distribution is defined below in equation (5).

$$\Pr(z_i = k | \mathbf{w}, \mathbf{t}, \mathbf{l}, \mathbf{z}_{-i}, \alpha^{(l)}, \beta^{(k)}, \mu^{(k)}, \gamma^{(k)}) \propto \frac{s_{w_i}^{k,-i} + \mu'}{\sum_{v=1}^{S}(s_v^{k,-i} + \mu')} \cdot \frac{m_{t_i}^{k,-i} + \gamma_{t_i}^{(k)}}{\sum_{t=1}^{T}(m_t^{k,-i} + \gamma_t^{(k)})} \cdot \frac{o_k^{l,-i} + \alpha_k^{(l)}}{\sum_{k'=1}^{K}(o_{k'}^{l,-i} + \alpha_{k'}^{(l)})} \cdot \pi_k \tag{4}$$

$$\Pr(z_i = k | \mathbf{w}, \mathbf{t}, \mathbf{l}, \mathbf{z}_{-i}, \alpha^{(l)}, \beta^{(k)}, \mu^{(k)}, \gamma^{(k)}) \propto \frac{n_{w_i}^{k,-i} + \beta'}{\sum_{v=1}^{V}(n_v^{k,-i} + \beta')} \cdot \frac{m_{t_i}^{k,-i} + \gamma_{t_i}^{(k)}}{\sum_{t=1}^{T}(m_t^{k,-i} + \gamma_t^{(k)})} \cdot \frac{o_k^{l,-i} + \alpha_k^{(l)}}{\sum_{k'=1}^{K}(o_{k'}^{l,-i} + \alpha_{k'}^{(l)})} \cdot (1 - \pi_k) \tag{5}$$

equations (3), (4) and (5), $n_{w_i}^{k,-i}$ denotes the number of times word w_i is assigned to regular topic k across all entries in N_l except the ith entry, $s_{w_i}^{k,-i}$ denotes the number of times seed word w_i is assigned to seed topic k across all entries in N_l except the ith entry, $m_{t_i}^{k,-i}$ denotes the number of times time point t_i is assigned to topic k across all entries in N_l except the ith entry and $o_k^{l,-i}$ denotes the number of times location l is associated with topic k across all entries in N_l except the ith entry. $\alpha_k^{(l)}$ refers to the kth component of $\alpha^{(l)}$ and $\gamma_{t_i}^{(k)}$ denotes the component of $\gamma^{(k)}$ corresponding to time point t_i.

Implementing the collapsed gibbs sampler. Collapsed gibbs sampler for the supervised topic model is surprisingly easy to implement. It involves setting up the required count variables, randomly initializing them, and then the gibbs sampler executes in an iterative fashion where on each iteration a topic is sampled for each entry in N_l according to equation (3) or equation (4) and equation (5) depending on whether the word in the entry is a regular word or a seed word respectively. The required count variables include $n_{w_i}^k$, $s_{w_i}^k$, $m_{t_i}^k$ and o_k^l corresponding to the ith entry in N_l. For simplicity and efficiency, we also keep a running count of n^k ($= \sum_{v=1}^{V} n_v^k$, the total number of times any word in vocabulary V is assigned to topic k), s^k ($= \sum_{v=1}^{S} s_v^k$, the total number of times any word in the set S of seed words is assigned to the corresponding seed topic k), m^k ($= \sum_{t=1}^{T} m_t^k$, the total number of times any time point $t \in \{1, 2, \cdots, K\}$ is assigned to topic k) and o^l ($= \sum_{k=1}^{K} o_k^l$, the total number of times any topic $k \in \{1, 2, \cdots, K\}$ is associated with location l). Finally, in addition to the mentioned count variables, we also require an array $\mathbf{z}$ which will contain the topic assignment for each entry or tuple in N_l. Once we choose a topic for a particular entry in N_l, the chosen topic is set in the $\mathbf{z}$ array and the count variables are incremented in the appropriate position relevant to the entry.

Following the gibbs iterations, the count variables can be used to compute the output (latent) parameters θ, ϕ^r , ϕ^s and ξ as shown below in equation (6).

$$\begin{aligned}
\theta_{l,k} &= \frac{o_k^l + \alpha_k^{(l)}}{\sum_{k'=1}^{K}(o_{k'}^l + \alpha_{k'}^{(l)})} \\
\phi_{k,w}^r &= \frac{n_w^k + \beta'}{\sum_{v=1}^{V}(n_v^k + \beta')} \\
\phi_{k,w}^s &= \frac{s_w^k + \mu'}{\sum_{v=1}^{S}(s_v^k + \mu')} \\
\xi_{k,t} &= \frac{m_t^k + \gamma_t^{(k)}}{\sum_{t=1}^{T}(m_t^k + \gamma_t^{(k)})}
\end{aligned} \tag{6}$$

where, $\theta_{l,k}$ represents the probability of topic k given location l, $\phi_{k,w}^r$ represents the probability of word w given topic k, $\phi_{k,w}^s$ represents the probability of seed word w given seed topic k and $\xi_{k,t}$ denotes the temporal trend value of topic k at time point t. We ran the gibbs sampler for 300 iterations.

Baseline methods for case count estimation

We compared EpiNews-ARNet with 2 baseline methods, namely Casecount-ARMA and EpiNews-ARMAX. In Casecount-ARMA, we fitted an autoregressive-moving-average model (ARMA(p, q)[16]) over past disease case counts to generate case count estimates as shown below in equation (7).

$$\hat{S}_{D,T} = \varepsilon_{D,T} + \sum_{i=1}^{p} \gamma_i S_{D,T-i} + \sum_{i=1}^{q} \theta_i \varepsilon_{D,T-i} \tag{7}$$

where, p and q are the orders of the autoregressive (AR) and moving average (MA) components, respectively. $\varepsilon_{D,T}, \varepsilon_{D,T-1}, \ldots, \varepsilon_{D,T-q}$ represent the white noise error terms. For further details including boundary conditions of ARMA, please refer to Box et al.[16] Casecount-ARMA doesn't use any information related to temporal topic trends (ξ_z). However, in EpiNews-ARMAX, we used an autoregressive–moving-average model with external input variables (ARMAX(p,q)[16]). As shown below in equation (8), ARMAX(p, q) incorporates information from both past case counts and temporal topic trends (ξ_z) in order to estimate case counts. Similar to EpiNews-ARNet, external input variables are represented by the temporal topic trends (ξ_z).

$$\hat{S}_{D,T} = \varepsilon_{D,T} + \sum_{i=1}^{p} \gamma_i S_{D,T-i} + \sum_{i=0}^{p} \eta_i \xi_{z,T-i} + \sum_{i=1}^{q} \theta_i \varepsilon_{D,T-i} \tag{8}$$

where, p and q are the orders of the autoregressive (AR) and moving average (MA) components, respectively. For further details, please refer to Box et al.[16]

References

1. Peters, M. E. & Lecocq, D. Content extraction using diverse feature sets. In *Proceedings of the 22nd International Conference on World Wide Web Companion*, 89–90 (International World Wide Web Conferences Steering Committee, 2013).
2. Webster, J. J. & Kit, C. Tokenization as the initial phase in nlp. In *Proceedings of the 14th Conference on Computational Linguistics*, vol. 4, 1106–1110 (Association for Computational Linguistics, 1992).
3. Singh, V. & Saini, B. An effective pre-processing algorithm for information retrieval systems. *International Journal of Database Management Systems* **6**, 13 (2014).
4. Kanis, J. & Skorkovská, L. Comparison of different lemmatization approaches through the means of information retrieval performance. In *Text, Speech and Dialogue*, 93–100 (Springer, 2010).
5. Ramakrishnan, N. *et al.* 'Beating the news' with EMBERS: Forecasting civil unrest using open source indicators. In *Proceedings of the 20th ACM SIGKDD International Conference on Knowledge Discovery and Data Mining*, 1799–1808 (ACM, 2014).
6. Doyle, A. *et al.* Forecasting significant societal events using the embers streaming predictive analytics system. *Big Data* **2**, 185–195 (2014).
7. Chakraborty, P. *et al.* Forecasting a moving target: Ensemble models for ili case count predictions. In *Proceedings of the 2014 SIAM International Conference on Data Mining*, 262–270 (SIAM, 2014).
8. Rekatsinas, T. *et al.* Sourceseer: Forecasting rare disease outbreaks using multiple data sources. In *Proceedings of the 2015 SIAM International Conference on Data Mining*, 379–387 (SIAM, 2015).
9. Blei, D. M., Ng, A. Y. & Jordan, M. I. Latent dirichlet allocation. *The Journal of Machine Learning Research* **3**, 993–1022 (2003).
10. Matsubara, Y., Sakurai, Y., Faloutsos, C., Iwata, T. & Yoshikawa, M. Fast mining and forecasting of complex time-stamped events. In *Proceedings of the 18th ACM SIGKDD International Conference on Knowledge Discovery and Data Mining*, 271–279 (ACM, 2012).
11. Rosen-Zvi, M., Griffiths, T., Steyvers, M. & Smyth, P. The author-topic model for authors and documents. In *Proceedings of the 20th Conference on Uncertainty in Artificial Intelligence*, 487–494 (AUAI Press, 2004).
12. Jagarlamudi, J., Daumé III, H. & Udupa, R. Incorporating lexical priors into topic models. In *Proceedings of the 13th Conference of the European Chapter of the Association for Computational Linguistics*, 204–213 (Association for Computational Linguistics, 2012).

Whooping cough topic		Rabies topic		Salmonellosis topic		E. coli infection topic	
Seed words		Seed words		Seed words		Seed words	
child	0.1498	animal	0.1596	food	0.2056	coli	0.2265
school	0.1068	rabies	0.1191	salmonella	0.1031	boil	0.0887
cough	0.0828	rabid	0.0718	product	0.1013	cell	0.0745
pertussis	0.0701	bite	0.0695	recall	0.0878	toxin	0.0628
whoop	0.0691	rabie	0.0674	drug	0.0712	escherichia	0.0617
whooping	0.0679	virus	0.0649	consumer	0.0705	clinical	0.0573
infant	0.0596	wild	0.0585	contamination	0.0598	chemical	0.0557
student	0.0557	bat	0.0472	fda	0.0579	kidney	0.0414
contagious	0.0454	raccoon	0.0471	contaminate	0.0567	microbiology	0.0402
booster	0.0406	skunk	0.0424	abdominal	0.0351	reaction	0.0397
cold	0.0395	fox	0.0422	egg	0.0277	hemolytic	0.0376
coughing	0.0309	wildlife	0.0379	chicken	0.0275	lettuce	0.0366
nose	0.0304	domestic	0.0323	poultry	0.025	uremic	0.036
respiratory	0.0284	saliva	0.0247	arthritis	0.0145	physical	0.0342
mild	0.0269	scratch	0.0237	peanut	0.0139	gene	0.0339
tdap	0.0231	quarantine	0.0213	cantaloupe	0.01	shiga	0.0202
immunize	0.0212	horse	0.0192	shell	0.0086	expression	0.0162
runny	0.0198	viral	0.0190	typhimurium	0.0083	chemistry	0.0149
tetanus	0.0175	livestock	0.0166	newport	0.0082	stec	0.0125
breathe	0.0144	mammal	0.0156	enteritidis	0.0074	biochemistry	0.0094
Regular words with higher probabilities		Regular words with higher probabilities		Regular words with higher probabilities		Regular words with higher probabilities	
contact	0.0037	pet	0.0037	eat	0.0019	transmit	0.0014
young	0.0023	contact	0.0037	diarrhea	0.0019	massachusetts	0.0013
adult	0.0022	cat	0.0028	nausea	0.0013	surface	0.0012
vaccination	0.0019	vaccination	0.0024	foodborne	0.0013	body	0.0012
vaccine	0.0019	florida	0.0015	package	0.0012	pennsylvania	0.0012
california	0.0019	vaccine	0.0014	contaminated	0.0011	blood	0.0012
vaccinate	0.0018	shot	0.0014	meat	0.0011	pathogen	0.0011
parent	0.0017	street	0.0013	restaurant	0.0010	resistant	0.0011
woman	0.0015	clinic	0.0012	vomit	0.0010	drinking	0.0011
baby	0.0014	texas	0.0010	products	0.0008	agricultural	0.0011
immunization	0.0011	park	0.0010	cook	0.0008	hygiene	0.0010
kid	0.0009	york	0.0010	beef	0.0008	raw	0.0009
air	0.0008	wound	0.0009	raw	0.0007	apple	0.0009
weather	0.0007	virginia	0.0008	temperature	0.0006	sandwich	0.0009
pregnant	0.0006	ferret	0.0007	honey	0.0005	milk	0.0008
mother	0.0006	brain	0.0007	pepper	0.0004	stool	0.0008
dose	0.0006	coyote	0.0005	weather	0.0003	parasite	0.0005
antibiotic	0.0005	nervous	0.0005	salad	0.0003	acs	0.0002
pneumonia	0.0003	canine	0.0002	mango	0.0002	receptor	0.0001

Table 1. Four disease topics (Whooping Cough, Rabies, Salmonella and E. coli infection) discovered by the supervised topic model from the HealthMap corpus for U.S. For each disease topic, we show the seed words and their corresponding probabilities in the seed topic distribution. Along with the seed words, we also show some of the regular words (having higher probabilities in the regular topic distribution) discovered by the supervised topic model related to these input seed words.

13. Rubin, T. N., Chambers, A., Smyth, P. & Steyvers, M. Statistical topic models for multi-label document classification. *Machine learning* **88**, 157–208 (2012).

14. Wallach, H. M., Mimno, D. M. & McCallum, A. Rethinking LDA: Why priors matter. In *Advances in Neural Information Processing Systems*, 1973–1981 (2009).

15. Griffiths, T. L. & Steyvers, M. Finding scientific topics. *Proceedings of the National Academy of Sciences* **101**, 5228–5235 (2004).

16. Box, G. E., Jenkins, G. M. & Reinsel, G. C. *Time series analysis: forecasting and control*, vol. 734 (John Wiley & Sons, 2011).

H7N9 topic		HFMD topic		Dengue topic	
Seed words		Seed words		Seed words	
flu	0.1229	hand	0.1573	fever	0.2269
bird	0.1225	child	0.1384	dengue	0.1586
avian	0.1053	mouth	0.1127	mosquito	0.1052
influenza	0.1051	school	0.1016	october	0.0826
human	0.1031	foot	0.0916	water	0.0682
virus	0.0832	class	0.0734	breeding	0.0559
poultry	0.0786	hfmd	0.0557	street	0.0481
market	0.0610	parent	0.0546	bite	0.0330
animal	0.0360	nursery	0.0343	aedes	0.0317
chicken	0.0303	kindergarten	0.0294	pain	0.0294
respiratory	0.0230	oral	0.0192	breed	0.0280
spring	0.0227	intestinal	0.0185	park	0.0269
farm	0.0224	infant	0.0178	sanitation	0.0179
farmer	0.0213	mumps	0.0174	borne	0.0175
slaughter	0.0194	measles	0.0172	albopictus	0.0168
winter	0.0179	herpes	0.0140	rain	0.0139
egg	0.0125	enterovirus	0.0135	hemorrhagic	0.0125
pandemic	0.0117	encephalitis	0.0124	vector	0.0115
h7n9	0.0012	dysentery	0.0117	larva	0.0089
h5n1	0.0000	ulcer	0.0093	aegypti	0.0066
Regular words with higher probabilities		Regular words with higher probabilities		Regular words with higher probabilities	
zhejiang	0.0034	shandong	0.0028	guangdong	0.0071
beijing	0.0034	hunan	0.0025	guangzhou	0.0056
shanghai	0.0030	care	0.0015	site	0.0013
agriculture	0.0015	rash	0.0008	temperature	0.0010
pneumonia	0.0013	meningitis	0.0007	weather	0.0009
temperature	0.0011	viral	0.0007	muscle	0.0008
food	0.0010	hepatitis	0.0007	blood	0.0006
eat	0.0009	body	0.0006	urban	0.0005
duck	0.0008	tuberculosis	0.0006	bleed	0.0004
pigeon	0.0008	childhood	0.0005	diarrhea	0.0004
cook	0.0006	palm	0.0004	medicine	0.0004
vaccine	0.0006	organ	0.0003	stagnant	0.0004
tamiflu	0.0005	skin	0.0003	spray	0.0003
meat	0.0004	buttock	0.0003	rainy	0.0003
strain	0.0004	childcare	0.0003	climate	0.0003
raw	0.0003	blister	0.0002	cough	0.0002
pig	0.0003	kidney	0.0002	tank	0.0002

Table 2. Three disease topics (H7N9, HFMD and dengue) discovered by the supervised topic model from the HealthMap corpus for China. For each disease topic, we show the seed words and their corresponding probabilities in the seed topic distribution. Along with the seed words, we also show some of the regular words (having higher probabilities in the regular topic distribution) discovered by the supervised topic model related to these input seed words.

ADD topic		Dengue topic		Malaria topic	
**Seed words**		**Seed words**		**Seed words**	
fall	0.1284	dengue	0.2090	malaria	0.1504
child	0.1148	fever	0.0978	mosquito	0.1166
school	0.0949	municipal	0.0759	site	0.0994
student	0.0868	breeding	0.0658	water	0.0893
food	0.0837	borne	0.0586	awareness	0.0826
consume	0.0611	mosquito	0.0555	lead	0.0735
eat	0.0588	september	0.0491	vector	0.0678
vomit	0.0549	august	0.0429	breed	0.0567
meal	0.0525	water	0.0408	monsoon	0.0484
stomach	0.0412	rain	0.0385	blood	0.0414
diarrhea	0.0315	aedes	0.0382	construction	0.0331
nausea	0.0304	ward	0.0382	camp	0.0316
vomiting	0.0300	platelet	0.0330	drug	0.0228
poisoning	0.0249	breed	0.0300	rainfall	0.0175
poison	0.0241	larva	0.0268	typhoid	0.0148
midday	0.0237	blood	0.0264	tribal	0.0133
contaminated	0.0183	bite	0.0246	falciparum	0.0114
cook	0.0179	chikungunya	0.0206	economic	0.0110
lunch	0.0117	vector	0.0199	anopheles	0.0099
contaminate	0.0105	monsoon	0.0084	plasmodium	0.0084
**Regular words with higher probabilities**		**Regular words with higher probabilities**		**Regular words with higher probabilities**	
village	0.0032	civic	0.0038	mumbai	0.0025
bihar	0.0023	delhi	0.0026	virus	0.0015
inflammatory	0.0020	virus	0.0018	maharashtra	0.0011
sample	0.0018	temperature	0.0014	stagnant	0.0011
odisha	0.0017	fogging	0.0014	insect	0.0009
ache	0.0011	haryana	0.0009	garbage	0.0008
sick	0.0010	spray	0.0009	flu	0.0008
pain	0.0008	stagnant	0.0008	spraying	0.0007
iron	0.0008	infection	0.0008	aegypti	0.0007
rice	0.0006	aegypti	0.0008	parasite	0.0006
pesticide	0.0005	drain	0.0007	tank	0.0006
flood	0.0004	larval	0.0006	leptospirosis	0.0005
drink	0.0004	stagnate	0.0005	urban	0.0004
sanitation	0.0004	gutter	0.0003	drainage	0.0003
stale	0.0003	rainwater	0.0002	rainwater	0.0002
drinking	0.0001	urbanization	0.0001	waterlog	0.0002

Table 3. Three disease topics (ADD, dengue and malaria) discovered by the supervised topic model from the HealthMap corpus for India. For each disease topic, we show the seed words and their corresponding probabilities in the seed topic distribution. Along with the seed words, we also show some of the regular words (having higher probabilities in the regular topic distribution) discovered by the supervised topic model related to these input seed words.
